# Anti-tuberculosis drug resistance in Slovakia, 2018–2019: The first whole-genome epidemiological study

**DOI:** 10.1016/j.jctube.2021.100292

**Published:** 2021-12-20

**Authors:** Matúš Dohál, Věra Dvořáková, Miluše Šperková, Igor Porvazník, Andrea Maurizio Cabibbe, Alberto Trovato, Andrea Spitaleri, Erik Michael Rasmussen, Kristián Pršo, Mária Škereňová, Daniela Maria Cirillo, Ivan Solovič, Juraj Mokrý

**Affiliations:** a Department of Pharmacology and Biomedical Center Martin, Jessenius Faculty of Medicine, Comenius University, Slovakia; b National Reference Laboratory for Mycobacteria, National Institute of Public Health, Praha, Czech Republic; c National Institute of Tuberculosis, Lung Diseases and Thoracic Surgery, Vyšné Hágy, Slovakia, Faculty of Health, Catholic University, Ružomberok, Slovakia; d Faculty of Health, Catholic University, Ružomberok, Slovakia; e Emerging Bacterial Pathogens Unit, IRCCS San Raffaele Scientific Institute, Milan, Italy; f International Reference Laboratory of Mycobacteriology, Statens Serum Institut, Copenhagen, Denmark; g Biomedical Center Martin, Department of Molecular Medicine, Jessenius Faculty of Medicine in Martin, Comenius University in Bratislava, Slovakia, Department of Clinical Biochemistry, Jessenius Faculty of Medicine in Martin, Comenius University in Bratislava, Slovakia

**Keywords:** Multi drug resistant, Extensively drug resistant, Tuberculosis, Slovakia, Whole genome sequencing

## Abstract

**Objective:**

The resistance of *Mycobacterium* (*M.*) *tuberculosis* to antituberculosis drugs poses a major threat to global public health. Whole genome sequencing (WGS) is an increasingly preferred method in the diagnostics and monitoring of the transmission dynamics of resistant forms of tuberculosis (TB). The aim of the study was to, for the first time, use the sequencing-based analysis to study the transmission and resistance patterns of a systematic and recent collection of extensively drug resistant (XDR) and multidrug resistant tuberculosis (MDR-TB) isolates and to expand our knowledge about drug resistant (DR) TB epidemiological dynamics in Slovakia.

**Design:**

A total of 495 patients with pulmonary TB, who were referred to National Reference Laboratory for Mycobacteriology (Vyšné Hágy, Slovakia) in the years 2018–2019, were studied. Out of the total of 495 patients, 4 XDR-TB (0.8%) and 8 (1.6%) MDR-TB isolates were identified by conventional drug susceptibility testing on Löwenstein-Jensen solid medium and subjected to whole genome sequencing. Sequencing data were evaluated for molecular-epidemiological analysis and identification of resistance patterns.

**Results:**

Phylogenetic and cluster analysis showed extensive recent transmission events and the predominance of Euro-American lineage 4.7 in Slovakia. However, phylogenetic analysis revealed the circulation of several lineages that originally occurred in Eastern European countries. Resistance patterns for first- and second-line antituberculosis drugs characterized by whole genome sequencing were in high concordance with the results of phenotypic drug susceptibility testing.

**Conclusion:**

Forty percent of at least MDR-TB isolates were not genetically linked, indicating that appropriate measures should be taken to monitor and prevent the spread of drug-resistant tuberculosis within the country as well as in other regions.

## Highlights

• The Euro-American lineage 4.7 was the most represented among the XDR-TB/MDR-TB resistant strains.
• Seven of 12 isolates (58%) belonged to the same recent transmission chain.
• Strains originally from Eastern European countries were diagnosed in patients without any epidemiological links with these countries.
• Incidence of resistant XDR-TB/MDR-TB in Slovakia, while low, still should be taken to monitor.

## 1 Introduction

In 2019, 214 cases (3.93/100,000 population) of diagnosed tuberculosis (TB) were reported in Slovakia, indicating a remarkable decrease compared to 2018 (281 diagnosed TB cases, 5.45/100,000 population) (ECDC, 2020). Despite the globally increasing incidence of resistant forms of TB, the number of reported multidrug resistant (MDR) (strains resistant to isoniazid and rifampicin) and extensively drug resistant (XDR) (MDR-TB strains additionally resistant to fluoroquinolones and second-line injectable drugs) TB cases in Slovakia is relatively stable: 2 XDR-TB / 7 MDR-TB cases in 2015, 8 MDR-TB cases in 2016, 3 XDR-TB / 10 MDR-TB cases in 2017, 2 XDR-TB / 3 MDR-TB cases in 2018 and 2 XDR-TB / 5 MDR-TB cases in 2019. Moreover, the success rate for the treatment of resistant forms of TB is among the highest in Europe [1] (National Register of Tuberculosis, Slovakia).

Performing systematic whole genome sequencing (WGS) enables rapid, reliable, and complex insight into the *Mycobacterium (M.) tuberculosis* genome linked to anti-TB drug resistance [2], and, in phylogenetic analysis, it allows to determine *M. tuberculosis* complex (MTBC) lineages, analysis of transmission events, and tracing TB outbreaks with a higher discriminatory power compared to traditionally used tools [3], [4], [5], [6].

Studies have shown that the individual lineages may differ in resistance profile, virulence, transmissibility and may also determine the clinical outcome of tuberculosis [7], [8]. The classification of *M. tuberculosis* lineages, as well as the genomic relatedness of individual strains, has not been performed in Slovakia by any molecular technique. The objective of this study is to describe the underlying resistance patterns and characterize the genomic variability of the MDR- and XDR *M. tuberculosis* strain using WGS technology. WGS data will provide a baseline set of major clones and lineages of resistant TB in this country and cluster analysis will contribute to the localization of outbreaks at the regional level.

## 2 Material and methods

### 2.1 Clinical isolates

In 2018 and 2019, 495 *M. tuberculosis* strains, from more than 49,000 samples collected, were reported at the National reference laboratory for mycobacteriology in Vyšné Hágy, High Tatras, Slovakia, which is the only centralized clinical microbiological laboratory dedicated to the diagnosis of TB in the country. The pulmonary form was confirmed in 423 cases (85.5%) and the extrapulmonary form (primarily affecting the bones and joints of the spine) in 72 cases (14.5%). HIV as an associated disease was present in 6 patients (1.21%).

The presence of *M. tuberculosis* in clinical samples was confirmed by smear positivity and culture positivity on Löwenstein-Jensen (LJ) solid medium and in BACTEC^TM^ MGIT^TM^ 960 System (Becton and Dickinson, Franklin Lakes, USA).

Epidemiological data on patients were obtained from National reference laboratory for microbiology in Vyšné Hágy, High Tatras, Slovakia, under special codes, used also for the identification of individual isolates in this study.

### 2.2 Drug susceptibility testing

GeneXpert MTB/RIF assay (Cepheid, Sunnyvale, USA) was performed to determine rifampicin resistance. Conventional phenotypic drug susceptibility testing (pDST) by proportion method on LJ was then performed for first-line (rifampicin, isoniazid, ethambutol) and second-line drugs (streptomycin, amikacin, capreomycin, kanamycin, ethionamide, levofloxacin, moxifloxacin, cycloserine) and used as a standard method to define XDR-TB and MDR-TB cases. In this study, we used critical concentrations of antituberculosis drugs according to WHO recommendations [9]. The critical concentrations were 0.2 mg/L for isoniazid, 40.0 mg/L for rifampin, 2.0 mg/L for ethambutol, 2.0 mg/L for levofloxacin, 2.0 mg/L for moxifloxacin, 4.0 mg/L for streptomycin, 30.0 mg/L for amikacin, 30.0 mg/L for kanamycin, 40.0 mg/L for capreomycin, 30 mg/L for cycloserine. The results were determined after three weeks of incubation at 37 °C. Drug susceptibility to pyrazinamide was performed in BACTEC^TM^ MGIT^TM^ 960 System with the critical concentration 100 mg/L. WGS was carried out for strains with confirmed phenotypic resistance to at least isoniazid and rifampicin (MDR).

### 2.3 Whole genome sequencing

The well-grown colonies of MDR- and XDR strains of *M. tuberculosis* were suspended from the LJ culture into 200 μL DNA/RNA free water and inactivated (95 °C for 30 min). The genomic DNA was extracted according to the manufacturer's protocol using QIAamp DNA Mini Kit (QIAGEN, Hilden, Germany). Before sequencing, DNA was quantified by Qubit 4.0 using the Qubit dsDNA HS Assay kit (ThermoFisher Scientific, Waltham, USA) and subsequently processed for WGS using the Illumina Nextera XT library preparation kit (Illumina, San Diego, United States) and 2x150bp paired-end reads sequencing chemistry Illumina MiSeq Reagent Kit v2 (300-cycles). Sequencing was performed on the Illumina MiSeq 500 platform producing paired FASTQ files for each sample.

### 2.4 Bioinformatics and data analysis

Sequencing data were analyzed using the MTBseq pipeline (v1.0.2) to identify all variants in the genomes and MTBC lineage [10]. The analysis was performed on the mapped MTBC reads by setting a quality threshold of a mean coverage of at least 20x and an unambiguous base call threshold of ≥75%. A mutation was called only if SNPs and/or indel variants were detected in at least eight reads (both forward and reverse reads) with a minimum Phred quality score of 20 and by considering a mutation frequency ≥75%. The regions of the MTBC H37Rv reference genome (GenBank accession number NC_000962.3). Cryptic [11], PhyResSe [12] and TB-profiler [13] databases were used to interpret gene mutations involved in resistance to first- and second-line antituberculosis drugs.

In cluster analysis, detected SNP positions with a reliable base call in at least 95% of the isolates and covered in all isolates were concatenated to a sequence alignment, excluding SNPs within a window of 12 base pairs from each other and those located in repetitive regions or resistance-associated genes. A distant matrix was generated from MTBseq and a minimum spanning tree was constructed using GrapeTree software with the maximum distance threshold of 5 SNPs for linked transmission [14], [15].

### 2.5 Core-genome multilocus sequencing typing (cgMLST)

cgMLST analysis was based on a comparison of sequencing data obtained in this study with sequencing data of 24 MDR resistant strains isolated in the Czech Republic from years 2018 and 2019. These data were provided by National Reference Laboratory for Mycobacteria at the National Institute of Public Health. Cluster analysis was conducted with the set maximum distance 5 allele variants using the cgMLST of 2,891 core genes implemented in Ridom SeqSphere^+^ software version 7.2.3 (Ridom© GmbH, Münster, Germany). A minimum spanning tree was built using the same software.

### 2.6 Ethics statement

This study was approved by the Ethics committee of Jessenius Faculty of Medicine in Martin (EK 72/2018), Comenius University in Bratislava, Slovakia.

### 2.7 Sequence data availability

WGS raw reads were submitted to the European Nucleotide Archive as FASTQ files under study accession no. PRJEB43174.

## 3 Results

### 3.1 Drug resistance profile concordance between phenotype and genotype

Based on the results of pDST, 483 isolates were confirmed to be fully susceptible to isonazid or rifampicin, 4 isolates were characterized as XDR and 8 isolates as MDR, which represented 0.8% and 1.6% of all diagnosed TB cases in Slovakia during the study period. Ten XDR-TB/MDR-TB cases were registered as new cases (83%) and two as relapse (17%). The results of pDST and genotypic drug susceptibility testing (gDST) were compared for all the samples and summarized in Table 1. Resistance to rifampicin, isoniazid and fluoroquinolones was determined in 100% agreement between pDST and gDST. Most frequently identified mutations were: *rpoB* Asp435Val for rifampicin (9/12), *katG* Ser315Thr for isoniazid (11/12), *embB* Met306Ile for ethambutol (9/11), *pncA* Leu182Ser for pyrazinamide (7/10), *rrs* 513c < t for streptomycin (6/9), *rrs* 1401a > g for aminoglycosides (2/3), *fabG* 15C > T for ethionamide (2/3), *gyrA* Asp94Gly for fluoroquinolones (6/9). One isolate (SVK257-19), did not harbor mutation encoding resistance to pyrazinamide and another isolate (SVK359-19) missed the mutation encoding resistance to streptomycin. In addition, no mutation encoding resistance to kanamycin was detected in two XDR-TB isolates (SVK210-19, SVK244-18). Moreover, all MDR-TB and XDR-TB isolates were sensitive to bedaquiline, clofazimine, and delamanide according to gDST results (susceptibility has not been tested by pDST for these drugs).

**Table 1:** Mutations identified in genes associated with resistance to first-line and second-line antituberculotics in MDR and XDR strains of *M. tuberculosis* occurring in Slovakia during 2018 and 2019.

Sample	RMP	INH	EMB	PZA	SM	AMG	ETA	FQ	BDQ	CFZ	LZD	DLM
SVK41-18**^C^**	*rpoB* p.Asp435Val	*katG* p.Ser315Thr	*embA* c.-16C > T *embB* p.Met306Ile	*pncA* p.Leu182Ser	*rrs r.513c < t	*rrs* r.1401a > g	S	*gyA* p.Asp94Gly	S	S	S	S
SVK45-18**^C^**	*rpoB* p.Asp435Val	*katG* p.Ser315Thr	*embA* c.-16C > T *embB* p.Met306Ile	*pncA* p.Leu182Ser	rrs * r.513c < t	S	S	*gyrA* p.Asp94Gly	S	S	S	S
SVK244-18**^C^**	*rpoB* p.Asp435Val	*katG* p.Ser315Thr	*embA* c.-16C > T *embB* p.Met306Ile	*pncA* p.Leu182Ser	*rrs r.513c < t	S by gDST R to kanamycin by pDST	S	*gyrA* p.Asp94Gly	S	S	S	S

SVK281-18**^C^**	*rpoB* p.Asp435Val	*katG* p.Ser315Thr	*embB* p.Met306Ile	*pncA* p.Leu182Ser	*rrs r.513c < t	S	S	*gyrA* p.Asp94Gly	S	S	S	S
SVK380-18	*rpoB* p.Ser 450 Phe	*katG* p.Ser315Thr	S by pDST *embB* p.Met306Val	*pncA* p.His71Asp	*rpsL* p.Lys43Arg	*eis* c.-37G > T	S	S	S	S	S	S
^A^SVK210-19**^C^**	*rpoB* p.Asp435Val	*katG* p.Ser315Thr	*embA* c.-16C > T *embB* p.Met306Ile	*pncA* p.Leu182Ser	*rrs r.513c < t	S by gDST R to kanamycin by pDST	S	*gyrA* p.Asp94Gly	S	S	S	S
SVK243-19	*rpoB* p.Gln432Lys	*furA_ups c.2156599_2156610del	S	S	S	S	S	S	S	S	S	S
SVK257-19	*rpoB* p.Asp435 Val	*katG* p.Ser315Thr	*embB* p.Gly406Cys	S by gDST R by pDST	*rpsL* p.Lys88Arg	S	S	S	S	S	S	S
SVK359-19	*rpoB* p.Ser450Leu *rpoC* p.Gly332Arg	*katG* p.Ser315Thr *fabG1* c.-15C > T	*embB* p.Met306Ile	*pncA* p.Ala146Val	S by gDST R by pDST	S	*fabG1* c.-15C > T *ethA* c.1391_1392insA	*gyrA* p.Ala 90Val *gyrA* p.Asp94His	S	S	*rplC p.Cys154Arg	S
SVK365-19**^C^**	*rpoB* p.Asp435Val	*katG* p.Ser315Thr	embB p.Met306Ile	*pncA* p.Leu182Ser	S	S	S	*gyrA* p.Ala 90Val	S	S	S	S
^B^SVK366-19**^C^**	*rpoB* p.Asp435Val	*katG* p.Ser315Thr	*embB* p.Met306Ile	*pncA* p.Leu182Ser	*rrs r.513c < t	S	S	*gyrA* p.Asp94Gly	S	S	S	S
SVK387-19	*rpoB* p.Asp435Val	*katG* p.Ser315Thr *fabG1* c.-15C > T	*embB* p.Met306Ile	*pncA* c.311_312insG	*gid* c.351_351del	*rrs* r.1401a > g	*fabG1* c.-15C > T	*gyrA* p.Asp94Tyr	S	S	S	S

RMP – rifampicin, INH – isoniazid, EMB – ethambutol, PZA – pyrazinamide, SM – streptomycin, AMG – aminoglycosides (kanamycin, amikacin, capreomycin), ETA – ethionamide, FQ – fluoroquinolones, BDQ – bedaquiline, CFZ – clofazimine, LZD – linezolid, DLM – delamanid, S – sensitive, R – resistant, * - low confidence mutation, ^A^ - retreatment patient SVK45-18, ^B^ - retreatment patient SVK281-18, gDST – genotypic drug susceptibility testing, pDST – phenotypic drug susceptibility testing, **^C^ -** clustered cases.

### 3.2 Lineage identification

**Fig. 1:**
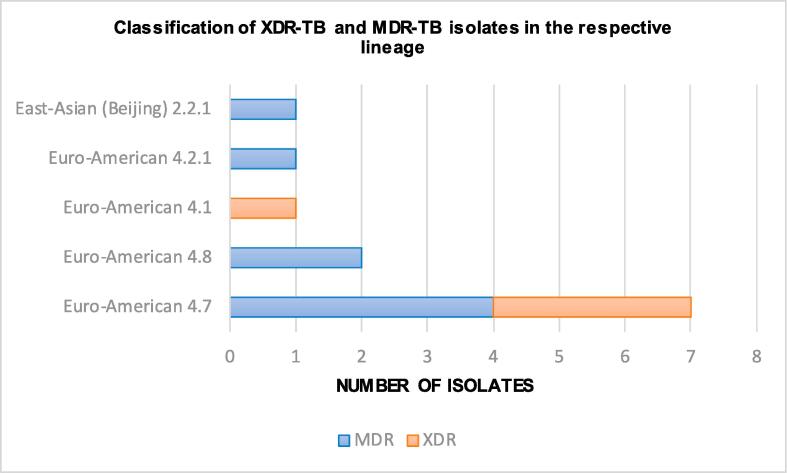
Classification of XDR-TB and MDR-TB isolates in the respective lineage.

A total of 11 out of 12 isolates (92%) belonged to Euro-American lineage 4. The 12 MDR *M. tuberculosis* strains were classified into 5 distinct sublineages. Our phylogenetic analysis revealed the dominant prevalence of Euro-American sublineage 4.7 (mainly T) among seven XDR- / MDR-TB isolates (58%) (Fig. 1, Table 2). Two isolates belonged to Euro-American sublineage 4.8, one isolate to the Ural genotype (Euro-American 4.2.1), one isolate to Beijing 2.2.1 sublineage (Europe/Russian W148 Outbreak), and one isolate to Euro-American sublineage 4.1.

**Table 2:** Epidemiologic data about XDR-TB and MDR-TB cases in Slovakia during 2018 and 2019.

Sample	Resistance	Cluster	Lineage	Patient information
SVK41-18	XDR	Clustered	Euro-American 4.7 (Mainly T)	Homeless community in Prievidza (a city in the midwest Slovakia)
SVK45-18	MDR	Clustered	Euro-American 4.7 (Mainly T)	Homeless community in Prievidza (a city in the midwest Slovakia)
SVK244-18	XDR	Clustered	Euro-American 4.7 (Mainly T)	Homeless community in Prievidza (a city in the midwest Slovakia)
SVK281-18	MDR	Clustered	Euro-American 4.7 (Mainly T)	Hospitalization at same time of patient 244–18
^A^SVK210-19	XDR	Clustered	Euro-American 4.7 (Mainly T)	Retreatment patient 45–18
SVK365-19	MDR	Clustered	Euro-American 4.7 (Mainly T)	The origin of the infection is not determined on the basis of available data
^B^SVK366-19	MDR	Clustered	Euro-American 4.7 (Mainly T)	Retreatment patient 281–18
SVK380-18	MDR	Non-clustered	Beijing 2.2.1 (Europe/Russian W148 Outbreak)	The origin of the infection is not determined on the basis of available data
SVK243-19	MDR	Non-clustered	Euro-American 4.8 (Mainly T)	The origin of the infection is not determined on the basis of available data
SVK257-19	MDR	Non-clustered	Euro-American 4.2.1 (Ural)	Patient originally from Ukraine
SVK359-19	MDR	Non-clustered	Euro-American 4.8 (Mainly T)	The origin of the infection is not determined on the basis of available data
SVK387-19	XDR	Non-clustered	Euro-American 4.1	The origin of the infection is not determinedon the basis of available data

^A^ - retreatment patient 45–18, ^B^ - retreatment patient 281–18.

### 3.3 Cluster analysis and detection of recent transmission chains

**Fig. 2:**
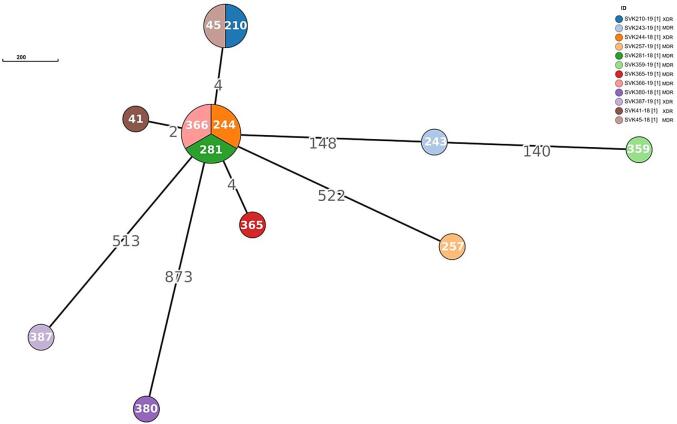
Minimum spanning tree based on SNP differences between the strains, including the XDR-TB and MDR-TB strains collected in Slovakia during 2018 and 2019. Maximum distance set to 5 SNPs for linked transmission. Distant matrix generated from MTBseq (version 1.0.2) and a minimum spanning tree was constructed using GrapeTree software.

In cluster analysis three XDR-TB, four MDR-TB (all belonged to the Euro-American sublineage 4.7) cases were identified within one cluster (transmission chain), based on determined SNPs, including two patients previously treated for TB (SVK45-18, SVK281-18) (Fig. 2). The distance between isolates within the cluster was 0–4 SNPs, indicating a recent transmission chain. Epidemiological investigation of clustered SVK45-18 (patient with retreatment TB SVK210-19), SVK244-18, and SVK41-18 confirmed that these patients lived together and shared the same spaces within the homeless community in a region of midwest Slovakia (Table 2). Also, the clustered patient SVK281-18 (patient with retreatment TB SVK366-19) was hospitalized at the same time as patient SVK244-18, indicating their epidemiological link (Table 2). Epidemiological data on the patient SVK365-19 did not confirm the link with other clustered isolates. Based on this discovery, we suggest that the recent transmission chain is not complete and some patients have not been diagnosed (Table 2).

cgMLST analysis did not define any of the isolates from Slovakia and Czech Republic within the same cluster, as these samples differed by more than 300 alleles.

## 4 Discussion

This study is the first documented in-depth WGS analysis of the molecular epidemiology and drug resistance patterns of XDR-TB/MDR-TB in Slovakia. A detailed description of lineages and clones represented will help to track resistant strains of *M. tuberculosis* internationally. We identified one larger cluster, indicating active transmission network and predominance of XDR-TB/MDR-TB strains of Euro-American lineage 4.7 spreading in Slovakia [16]*.* Based on epidemiological data; MDR-TB strain began to spread to various regions from the homeless community in western Slovakia. Moreover, our results confirmed the spread of this resistant strain among patients hospitalized at the same time, it will be therefore important for us to correlate the genetic relationship of the strains with the time of hospitalization. We assume that the XDR strain of Euro-American lineage 4.7 evolved from the MDR strain, probably after multiple and prolonged exposure to antibiotics or non-compliance with treatment [17]. Among XDR-TB isolates, only patient SVK387-19 was not defined as part of the recent transmission chain, as it deviated by more than 500 SNPs from the closest genome (Fig. 2). Regarding this isolate (from the physically disabled patient in contact with the family with negative TB history and caretaker aiding with activities of daily living), we were unable to clarify the origin of this strain. Due to frequent cross-border travel, we tried to identify the common ancestor strain of this XDR-TB isolate and other MDR-TB isolates from this study by the cgMLST in comparison with the WGS data from the Czech Republic (including MDR strains from years 2018–2019). cgMLST analysis confirmed no close relationship with any isolate from this database, as the difference between the samples from Slovakia and the Czech Republic was more than 12 alleles. In addition, four MDR isolates did not belong to any of the clusters determined in this study as they were more than 140–873 SNPs away from the closest related strain, indicated imported cases (Fig. 2).

Epidemiological data showed that most of the MDR-TB cases were diagnosed in Slovak patients. Only one patient (SVK380-18) with MDR-TB was born outside the Slovak Republic, more specifically in Ukraine. This patient was infected by Beijing sublineage 2.2.1, subgroup Europe/Russian W148 outbreak. Recent studies indicate that this subgroup is widespread in Ukraine among MDR-TB and XDR-TB isolates, suggesting the origin of the infection in this patient [18]. In general, Beijing strains are highly virulent and mobile, and their prevalence among resistant strains of *M. tuberculosis* will be the goal of further study. One isolate (SVK257-19) belonged to the Ural genotype (Euro-American 4.2.1), suggesting the partial spread of this MDR genotype beyond the borders of the countries of the former Soviet Union [19].

Lineage identification based on WGS data is in accordance with a recent study under the auspices of the European Centre for Disease Prevention and Control (ECDC) which confirmed that the Euro-American superlineage represents 75% among MDR-TB strains in Slovakia. Interestingly, the data reported by the ECDC revealed the prevalence of resistant strains of Beijing sublineage 2.2.1 circulating in the Czech Republic [20]. These results confirmed no transmission events of XDR-TB/MDR-TB strains, despite frequent cross-border travel between Slovakia and the Czech Republic.

In this study, the WGS uncovered 8 patients harboring MDR-TB isolates and 2 patients harboring XDR-TB isolates, which were also detected by phenotypic DST at National reference laboratory for mycobacteriology. Moreover, the clustered isolates shared the same mutation encoding resistance – to rifampicin (*rpoB* Asp435Val), isoniazid (*katG* Ser315Thr), ethambutol (*embB* Met306Ile), pyrazinamide (*pncA* Leu182Ser), and in 6/7 isolates the *gyrA* Asp94Gly mutation encoding resistance to fluoroquinolones (Table 1). were found. MDR-TB isolate (SVK365-19) was part of the cluster but showed a different mutation associated with resistance to fluoroquinolones (*gyrA* p.Ala90Val). This case most likely shared the common Euro-American lineage 4.7 MDR-TB ancestral strain circulating in Slovakia and the evolving of different mutation could be probably related to the non-adherence to the treatment regimen. In addition, 4/7 (57%) clustered isolates possessed also the mutation in *embA* 16C > T encoding resistance to ethambutol.

No variation in *katG* and *inhA* genes was detected in isolate SVK243-19, considering the genomic deletion (12 bp) in the upstream variant of *furA* gene as potentially encoding isoniazid resistance (Table 1.) [21]. Low-confidence mutation in *rplC* gene (Cys154Arg) associated with linezolid resistance was identified in MDR-TB isolate SVK359-19 [22]. Two XDR-TB isolates (SVK244-18, SVK210-19) had a resistance pDST results for kanamycin (retested 2 times) but did not show any mutation (frequency 75%, 40% and 10% were studied) in *rrs*, *eis* and *tlyA*, indicating that resistance is caused by an unknown mutation. Moreover, SVK210-19 (retreatment patient SVK45-18) is an example of microevolution from being susceptible to resistant to kanamycin over 12 months. For ethambutol, one isolate having the mutation *embB* Met306Val had susceptible pDST results; which can be explained by the fact that this mutation could increase ethambutol MIC levels [23]. The greatest mutation variability among unclustered samples was observed for pyrazinamide, probably related to geographically different regions from which the isolates were obtained [24].

Using WGS, we characterized for the first time the complete resistance profile of XDR-TB/MDR-TB isolates in Slovakia, as only data obtained from Geno Type MTBDRplus, version 2.0 (Hain Lifescience GmbH, Nehren, Germany) were currently available [25]. The most frequent mutations identified in this study are in accordance with worldwide studies on MDR-TB/XDR-TB [26].

In overall, our results showed the recent transmission chain of XDR-TB/MDR-TB strains in Slovakia and the circulation of diverse MDR lineages that originally occurred in Western European countries in the last decade of the 20th century, presumably representing imported cases [27]. It should be stated that a significant proportion of patients with TB in Slovakia (more than 30%) belong to marginalized groups of the population, which significantly complicates the efforts of public health to reduce transmission and monitor compliance with the treatment regimen. For this reason, 288 field workers were covering 314 localities in the poorest and most endangered parts of Slovakia to increase awareness of TB in 2019. Also, to ensure adherence to the treatment regimen, each patient is treated under directly observed therapy for the first 6 weeks after being diagnosed with the disease. The analysis of data from 2018 revealed that the treatment regimens are fully indicated and correct, as we have up to 86% treatment success rate in newly diagnosed microscopically positive cases. However, the findings of our study indicate that the transmission of DR-TB in Slovakia is not completely under control and appropriate measures should be taken to monitor and prevent the spread of drug-resistant tuberculosis both within and outside of the country.

## 5 Conclusion

This study highlights the utility of performing WGS as an approach with high discriminatory power in identification of the resistance patterns and genomic relatedness of MDR-TB and XDR-TB strains circulating in Slovakia. The results showed the predominance and increased clustering rate of strains belonging to the Euro-American lineage 4.7. In addition, based on a combination of sequencing data and epidemiological information, we identified the nosocomial origin of MDR-TB in one patient within the cluster. These findings emphasize the importance of the application of this method in clinical practice where it would significantly contribute to the deployment of new anti-TB drug regimens and surveillance activities.

## 6 Limitation of the study

The main limitation of the study is the small sample size, as only strains from the years 2018 and 2019 could be obtained from the reference laboratory due to limited technical and human resources. Molecular genetic analysis with a larger collection of resistant isolates would provide a more detailed insight into evolutionary relationships among strains.

## Declaration of Competing Interest

The authors declare that they have no known competing financial interests or personal relationships that could have appeared to influence the work reported in this paper.
